# Mitochondrial DNA variation in the Italian Heavy Draught Horse

**DOI:** 10.7717/peerj.8996

**Published:** 2020-05-15

**Authors:** Hovirag Lancioni, Irene Cardinali, Andrea Giontella, Maria Teresa Antognoni, Arianna Miglio

**Affiliations:** 1 Department of Chemistry, Biology and Biotechnology, University of Perugia, Perugia, Italy; 2 Department of Veterinary Medicine, University of Perugia, Perugia, Italy

**Keywords:** Mitochondrial DNA variation, Italian Heavy Draught Horse, Horse mitochondrial haplogroup, Phylogeny, Management of genetic resources, Genetic conservation strategy

## Abstract

**Background:**

In the last decades, Italy as well as other developed countries have registered a decrease in the population size of many local horse breeds. The continuous crossbreeding has determined the dilution of genetic heritage of several native breeds. The Italian Heavy Draught Horse (IHD) is the only autochthonous Italian coldblooded horse among these breeds; therefore, it represents a resource to be preserved. In 1927, the first generation of this breed was officially created by crossing different Heavy Draught horses with local mares and recorded in a Studbook.

**Methodology:**

To provide the first comprehensive overview of the genetic diversity of Italian Heavy Draught horses from Central Italy, we produced and phylogenetically analysed 52 mitochondrial DNA (mtDNA) control-region sequences. Furthermore, we evaluated data available from GenBank (*N* = 568) to have a more complete scenario and to understand the relationships with other European Heavy Draught horse breeds.

**Results:**

Among the IHD samples that were analysed, we identified ten of the 17 haplogroups described in modern horses. Most of these sequences fell into L, G, and M lineages, thus showing the overall mtDNA legacy of the ancestral mares that were probably used at the initial stages of breeding selections a long time ago. The high mitochondrial haplotype diversity (Hd = 0.969) found in our samples reflected the multiple maternal origins of the horses. Our results highlighted a considerable percentage of haplotypes shared especially with Bardigiano and Hungarian Heavy Draught breeds. Furthermore, both the presence of four unique haplotypes detected in our samples and their absence among all equine mitochondrial published data demonstrate a mitochondrial peculiarity that needs to be further investigated and preserved with careful breeding practices.

## Introduction

The development of mechanization focused on animal productivity has drastically changed the management of different species leading the current animal husbandry to take care of high-productivity breeds paying special attention to selection programs. Local native breeds have been subjected to a selective crossbreeding in order to preserve their peculiarities (Bruford et al., 2015). Among the other species, *Equus caballus* is certainly one of the most investigated because of its strict link with human activities. Thanks to selection programs and crossbreeding processes, a great variety of horse breeds developed all over the world, especially for farming, transport, and military purposes.

In the last decades in Italy, as well as in many other developed countries, the traditional uses of horses have been abandoned, and many breeds have registered a decrease in population size (Pieramati et al., 2013; Cappelli et al., 2015). One of these breeds is the Italian Heavy Draught Horse (IHD), created by the Italian government for the Italian Army. This breed represents the most important autochthonous coldblooded horse in Italy (Bigi & Zanon, 2008). Its selection began in 1860 in a military stud of Ferrara and developed by crossing native mares of Po Valley with the large Belgian stallions. Therefore, to make the breed lighter and faster, Percheron and Breton bloods were added. At a later stage, the breeding area vastly extended to the Centre and South of Italy. Holder of the Studbook, the National Association of Italian Heavy Draught Horse (ANACAITPR, http://www.anacaitpr.it) reports that at the beginning of 20th century, the mares resulting from this selection program were crossed with Norfolk-Breton stallions, officially creating the first generation of Italian Heavy Draught Horse recorded in a Studbook in 1927. Thereafter, the French Breton was used to improve the heavy draft abilities of this breed. These crossings produced bulky yet also agile horses, with the adults weighing between 700 and 900 kg and the preferred withers height between 156 and 162 cm for males and 153–160 cm for females, which made this breed also perfect for farming. Thanks to the remarkable development and to the milking attitude of the mares, foals can reach a weight of 400 kg at 7–8 months. Even though the Italian Heavy Draught Horse is particularly adapted for agricultural work with minimum environmental impact, the improvement of mechanization reduced the use of these horses, and they became particularly suitable for meat production especially thanks to their fast growth speed and muscle mass development (Mantovani, Sartori & Pigozzi, 2013; Miglio et al., 2019a; Miglio et al., 2019b; Miglio et al., 2019c). At the end of 2017, the registered population size was 7,474 with the largest numbers in Lazio, Umbria, and Abruzzo regions (http://www.anacaitpr.it).

Over time, both the increasing productivity and the continuous crossbreeding have determined the dilution of autochthonous genetic heritage; in the recent past, the Italian Heavy Draught horses were involved in a demographic decline becoming at risk of extinction. Nowadays, concerning the risk of losing its genetic peculiarities, this breed belongs to the “vulnerable” category (www.fao.org/dad-is). In this scenario, where phenotypic traits and genealogical data are often insufficient to ascertain horse history and origins, morphometrical approach and traditional breeding strategies are supported by molecular analyses that provide a needful and reliable tool for an efficient management of genetic resources (Pieramati et al., 2011; Solé, Valera & Fernández, 2018). In the last decade, several research activities have focused on describing and preserving the genetic peculiarities and the biodiversity of local horse breeds through the analysis of uniparental markers or whole genomes (Leroy et al., 2009; Achilli et al., 2012; Silva et al., 2012; Brandariz-Fontes et al., 2013; Cardinali et al., 2016; Gemingguli et al., 2016; Ianella et al., 2017; Csizmár et al., 2018; Kakoi et al., 2018).

In particular, the Italian Heavy Draught breed was previously investigated in studies based on microsatellite markers (Bigi & Perrotta, 2012) and mitochondrial control region (Bigi, Perrotta & Zambonelli, 2014) compared to other Italian “heavy” horses. In these studies, the Italian Heavy Draught Horse clustered together with the “heavier” Italian horses (Bardigiano, Catria, and Haflinger) and was characterized by the highest haplotype and nucleotide diversity. Furthermore, a Bayesian approach showed the common origin between Catria, Maremmano, and Italian Heavy Draught breeds (Bigi & Perrotta, 2012).

Nevertheless, a comparison with those breeds employed for the creation of these horses and the other European Heavy Draught horses is still lacking.

For the first time, this study characterized the genetic diversity of Italian Heavy Draught horses from Central Italy through the analysis of the mitochondrial DNA (mtDNA) aiming to understand its maternal origins and the relationships with other continental European heavy horse breeds. The knowledge of genetic variability of the maternal founding lines represents an indispensable requirement both for the productive performance and the enhancement of autochthonous genetic resources (Giontella et al., 2019). Historical evidence shows that the Italian Heavy Draught Horse is the result of a long selection process although to date there are no genetic investigations on a considerable sample of this equine breed from Central Italy. In this context, our study aims to highlight its genetic peculiarities in order to preserve biodiversity by performing management and conservation programs.

## Materials & Methods

Samples consisting of 1,2 ml of peripheral blood were collected from 81 specimens belonging to Italian Heavy Draught (IHD) horse breed in Central Italy. When the genealogical information was available, we traced back to a maximum of three generations. The Italian name of the breed is CAITPR (Cavallo agricolo italiano da tiro pesante rapido). All experimental procedures were reviewed and approved by the Animal Research Ethics Committee of the University of Perugia (Comitato Universitario di Bioetica Prot. n. 2017-01) in accordance with the European Union Directive 86/609.

Total DNA was extracted by following manual (DNeasy^®^ Blood & Tissue Kit, Qiagen) or automated (MagCore^®^Automated Nucleic Acid Extractor) extraction protocols. Only for maternally unrelated individuals the mitochondrial control region was amplified and sequenced by referring to the protocols reported in Cardinali et al. (2016).

All the sequences (610 bps from np 15,491 to np 16,100) were assembled and aligned to the Equine Reference Sequence (ERS, NC_001640.1, Xu & Arnason, 1994) using Sequencher™ 5.10 software (Gene Codes Corporation) in order to visualize electropherograms and register any mutational differences. After mitochondrial haplotype identification, all the mtDNA control regions were classified in haplogroups according to the respective mutational motifs by referring to Achilli et al., (2012) (Table S1).

In order to have a more complete scenario on Italian and other European Heavy Draught horses, in the analyses, we included data available from GenBank belonging to different breeds for a total of 568 sequences: Bardigiano (BAR; *N* = 66), Belgian Draught (BED; *N* = 13), Breton (BRE; *N* = 3), Croatian Coldblood (CRC; *N* = 12), Franches-Montagnes (FRM; *N* = 31), Haflinger (HAF; *N* = 41), Hungarian Draught (HUD; *N* = 286), Italian Heavy Draught (IHD; *N* = 27), Lithuanian Heavy Draught (LHD; *N* = 3), Noriker (NOR; *N* = 10), Percheron (PER; *N* = 7), Polish Heavy Draught (PHD; *N* = 3), Rhineland Heavy Draught (RHD; *N* = 26), Vladimir Heavy Draught (VHD; *N* = 21), and Vyatskaya (VYA; *N* = 19) (Table S2).

The phylogenetic relationships among haplotypes were visualized through the construction of two median-joining trees using Network 10.0 (https://www.fluxus-engineering.com/). The first network was constructed by also considering data from other published Heavy Draught breeds by uniforming the reading range (from np 15,532 to np 15,738) for a total of 620 sequences. The second network included all the control-region sequences of IHD horses from this study (*N* = 52) and those available from GenBank (*N* = 27) (Bigi, Perrotta & Zambonelli, 2014).

Different mtDNA sequence variation parameters were estimated by using DnaSP 5.1 software (Librado & Rozas, 2009).

Pairwise Fst calculations were carried out using the Arlequin v3.5 software package (Excoffier, Laval & Schneider, 2005), and intra- as well as inter-population comparisons were performed based on the number of pairwise differences between sequences and figured using an Arlequin integrated R script (http://www.cran.r-project.org/).

In order to graphically display (and summarize) the mitochondrial relationships among the analysed samples, Principal Component Analyses (PCA) were performed using Excel software implemented by XLSTAT, as previously described (Cardinali et al., 2016; Di Lorenzo et al., 2018).

## Results and Discussion

The 81 IHD samples were collected from different farms in Umbria (55), Latium (20), and Abruzzo (7) regions. When available, we accurately evaluated their pedigree and excluded the maternally related individuals, thus the final dataset was represented by a total of 52 specimens (43 females and 9 males) (Table S1).

The analysis of 610 base pairs from the control-region sequences showed a high haplotype diversity (Hd = 0.969) with a total of 30 haplotypes and 53 polymorphic sites (S) detected (Table 1). Overall, by also including the other IHD horses available from GenBank, thus considering a shorter range (nps 15,491–15,958), a total of 45 haplotypes were observed (Table 1). This result confirmed a varied genetic structure for this breed as previously reported by a study based on microsatellite markers (Maretto & Mantovani, 2009).

**Table 1:** Estimates of genetic diversity for all breeds here considered. For breeds with less than four samples, the Tajima’s average number of nucleotide differences was not calculated.

**Breed**	**Country of origin**	**Breed Code**	**Range (nps)**	**N**	*π*	**Nh**	**Hd**	**S**	**k**
Italian Heavy Draught (from this study)	Italy	IHD ts	15,491–16,100	52	0.021	30	0.969	53	12.758
All Italian Heavy Draught horses	Italy	IHD	15,491–15,958	79	0.023	45	0.979	54	10.709
All Italian Heavy Draught horses	Italy	IHD	15,532–15,738	79	0.109	40	0.970	30	5.791
Bardigiano	Italy	BAR	15,532–15,738	66	0.106	23	0.933	25	5.603
Belgian Draught	Belgium	BED	15,532–15,738	13	0.085	6	0.859	14	4.487
Breton	France	BRE	15,532–15,738	3	0.151	3	1.000	12	NC
Croatian Coldblood	Croatia	CRC	15,532–15,738	12	0.089	11	0.985	15	4.727
Franches-Montagnes	France	FRM	15,532–15,738	31	0.095	14	0.867	18	4.748
Haflinger	Italy	HAF	15,532–15,738	41	0.105	22	0.949	28	5.568
Hungarian Draught	Hungary	HUD	15,532–15,738	286	0.111	55	0.955	34	5.791
Lithuanian Heavy Draught	Lithuania	LHD	15,532–15,738	3	0.138	3	1.000	11	NC
Noriker	Italy	NOR	15,532–15,738	10	0.095	6	0.889	15	5.044
Percheron	France	PER	15,532–15,738	7	0.102	4	0.810	12	5.429
Polish Heavy Draught	Poland	PHD	15,532–15,738	3	0.113	3	1.000	9	NC
Rhineland Heavy Draught	Germany	RHD	15,532–15,738	26	0.111	17	0.932	24	5.865
Vladimir Heavy Draught	Russia	VHD	15,532–15,738	21	0.108	14	0.948	24	5.733
Vyatskaya	Russia	VYA	15,532–15,738	19	0.075	10	0.912	15	3.988
**Total**			**15,532–15,738**	**620**	**0.030**	**120**	**0.969**	**49**	**5.776**

**Notes.**

N number of samples *π* nucleotide diversity Nh number of haplotypes Hd haplotype diversity S number of polymorphic sites k Tajima’s average number of nucleotide differences

In order to highlight the relationships with some European Heavy Draught breeds, we performed a further DnaSP analysis by including other published mitochondrial data. After uniforming the sequence range (nps 15,532–15,738) to allow the comparison between all samples, we obtained a total of 120 haplotypes, with 49 polymorphic sites (excluding gaps and ambiguous sites) and a Nei’s nucleotide diversity (*π*) of 0.030 (Table 1). The Tajima’s average number of nucleotide differences (k) between two randomly chosen sequences was 5.776.

IHD showed nucleotide diversity and Tajima’s indices on average for all breeds (0.11 and 5.8, respectively). As for the haplotype diversity, without considering the breeds represented by only three animals per breed (Breton, Lithuanian Heavy Draught, and Polish Heavy Draught), the highest value for the haplotype diversity (Hd>0.96) was found in Italian Heavy Draught and Croatian Coldblood which is the most numerous among the autochthonous draught horses originating from Croatia.

It is thought that CRC was created by crossing Croatian native warmblood mares with Noriker stallions under a significant influence of the English Thoroughbred and the Belgian Coldblood. Nowadays it is bred in central and north-western Croatia and is characterized by a great adaptability to maintenance conditions (Čačić, 2009; Ivanković et al., 2009). A study focusing on population structure and admixture analyses in three Draught Horse breeds (Austrian, Croatian and German) indicated that the recent change of administrative states could represent an important factor influencing the differentiation among Draught horses (Druml et al., 2007).

Furthermore, our analysis showed the lowest values for haplotype diversity in Belgian Draught, Franches-Montagnes, Noriker, and Percheron, giving evidence of a reduced variability in their partial mtDNA control regions. This finding could be due to the strict selection that involved these breeds, as they were used for crossbreeding and improving different local horses. Together with HAF, they are currently distributed in different continents (http://www.fao.org/dad-is/transboundary-breed). In particular, BED has played a considerable role in the development of other heavy breeds, and the foundation stock that originated from Belgium was originally known as the Brabant (www.agraria.org). In the early 20th century, the mares continued to look like the original Belgian type, and then they changed from the “working” to the “heavy” type (http://www.palm.be). NOR is considered to be indigenous to the central Alpine region of Europe, and its agricultural use started during the industrialisation period in the 20th century. By the time of the Middle Ages, a small, heavy, and compact horse had already been developed within the NOR breed, and it is currently considered one of the indigenous horses “of limited distribution” (http://www.aia.it). HAF developed from the original native Tyrolean ponies in Austria and Northern Italy (in the South Tyrol region) during the late 20th century. Since it was largely used to improve several local horses, it achieved the aim of a pure breed, and its standards are controlled by the World Haflinger Federation. PER appears to derive from ancient native horses originating from the Perche district, in France; nowadays it is mainly used for draft work, meat, and sport (http://www.fao.org/dad-is). In the early 20th century, PER was the most popular draft horse with the largest breed registry up to the New Continent.

Among the 26 haplotypes belonging to the 52 IHD samples here analysed, only four (15.38%) were unique (found in only one sample), one (3.85%) was shared between two IHD samples, and 21 (80.77%) were shared with other breeds (Table S3). If compared to the frequencies found in the other breeds, this latter value not only could be considered high but also it outlines the notable genetic variability typical of IHD that should be preserved. Overall, among the 120 haplotypes retrieved in the entire dataset, 62 (51.67%) were unique, 18 (15.00%) were shared within the same breed, and 40 (33.33%) were shared between different breeds.

**Figure 1:**
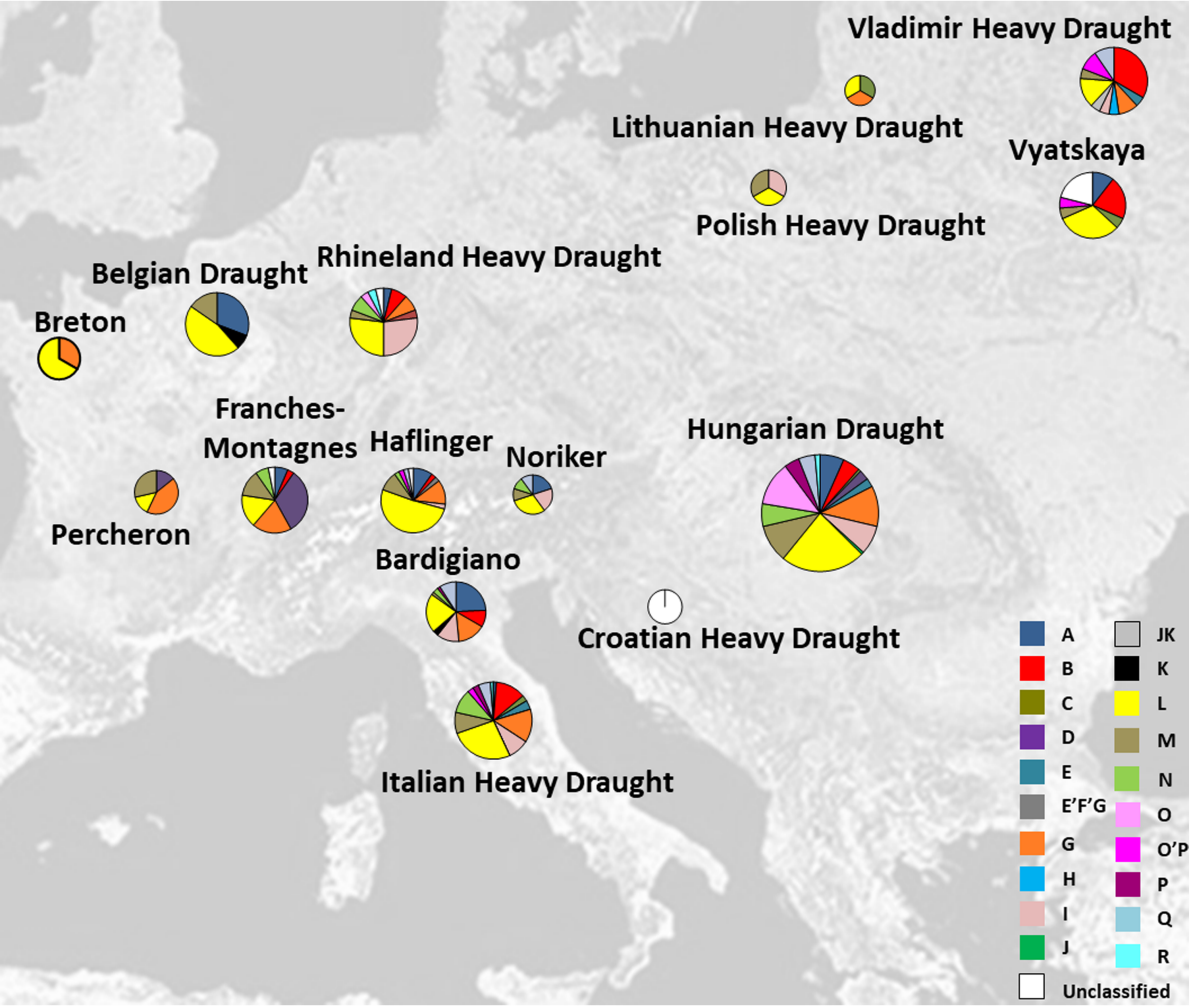
Italian and other European Heavy Horse breeds here analyzed and their respective haplogroup distribution. Pie charts are proportional to sample size (for details see Table 1). The map was modified from http://www.it.m.wikipedia.org/wiki/File:Europe_satellite_orthographic.jpg.

The analysis of mutational motifs identified so far (Achilli et al., 2012) has permitted the classification of all our haplotypes into ten haplogroups (B, C, E, G, I, L, M, N, P, and Q) (Table S4). As previously reported for most Italian breeds (Cozzi et al., 2004; Pieragostini et al., 2005; Maretto & Mantovani, 2009; Zuccaro et al., 2009; Felicetti et al., 2010; Guastella et al., 2011; Bigi & Perrotta, 2012; Bigi, Perrotta & Zambonelli, 2014; Morelli et al., 2014; Cardinali et al., 2016), 15 out of our 52 samples (29%) fell into L lineage, followed by G, M, and I (19%, 12%, and 10%, respectively). For the majority of the European heavy horse breeds that were considered in this study, the haplogroup L was the most common and widespread among all breeds as shown in Fig. 1 (see also Table S4).

The network analysis of all mitochondrial control regions belonging to the IHD from this study and those from Bigi, Perrotta & Zambonelli (2014) showed both a high number of unique haplotypes within our sample and no sequences belonging to haplogroup A (Fig. S1). Furthermore, among our samples, the most represented haplotypes belonged to G, M, and B haplogroups (HT13 found in eight samples, HT24 found in five samples, and HT26 found in four samples, respectively) (Table S1; Fig. S1). These haplotypes were shared with the following different European Heavy Draught Horse breeds (Table S2): HT13 was found also in FRM, HUD, LHD, PER, and RHD; HT24 was shared with BAR, BED, FRM, HAF, HUD, NOR, PHD, and RHD; HT26 was detected also in BAR, HAF, HUD, IHD from Bigi, Perrotta & Zambonelli (2014), RHD, VHD, and VYA.

**Figure 2:**
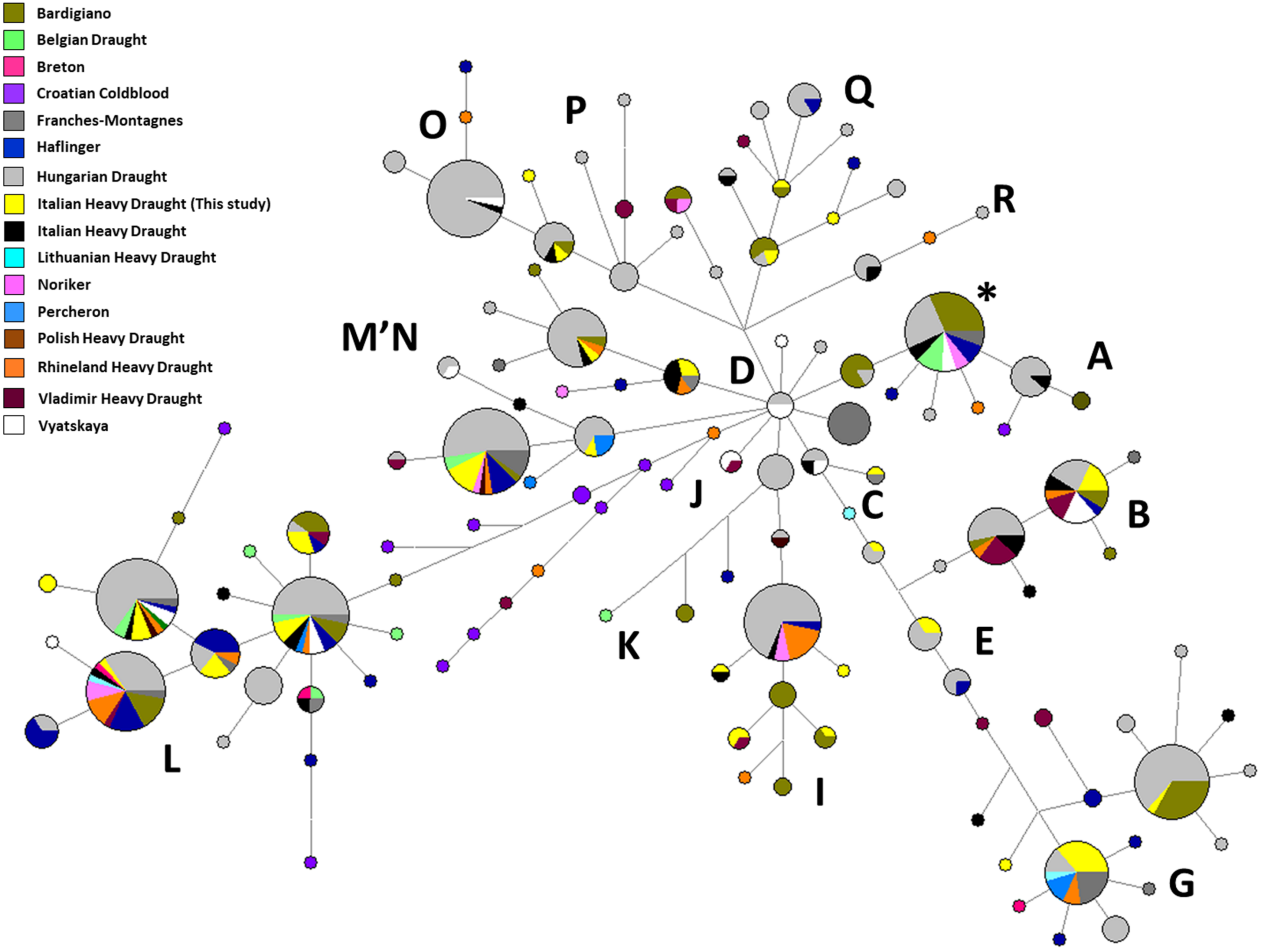
Median-Joining Network based on control-region sequences of Italian and other European Heavy Horse breeds here considered. The asterisk indicates the haplotype identical to ERS (Equine reference sequence; NC_001640.1).

The second network showed a high percentage of our haplotypes shared above all with both BAR and HUD followed by RHD (Fig. 2). BAR is widely distributed in Italy, and because of its relatively large numbers, this breed is not considered at risk of extinction; however, it is classed as “vulnerable” (Sabbioni et al., 2005). Together with HAF, BAR originated from crossing northern horses with local mares during the invasions in Roman period. On the other hand concerning HUD, it is reported that heavy draught horse breeds were not present in Hungary before their importation. This breed is the results of crossing local mares and stallions coming from the west (Csizmár et al., 2018).

Rhenish German Coldblood (RHD) originated from the Rhineland area of western Germany and developed in the 19th century by interbreeding local working horses with other draught horses from neighbouring countries. Then, due to the political division of Germany, gene flows between breeds from East Germany and the RHD were impossible for four decades; as a result of this reproductive isolation, a significant genetic differentiation between RHD and the East German horses was observed (Aberle et al., 2004; Aberle et al., 2007).

As mentioned above, the haplotype with the highest frequency among our samples (HT13, found in eight sequences out of 52, 15.40%) was shared with different European breeds. In the second network, we noticed five haplotypes found only in our samples which were not shared with the other breeds here considered (HT1, HT8, HT14, HT17 and HT18) (Fig. 2). Those novel haplotypes belong to different haplogroups, mainly haplogroup G, I, L, P and Q. Then, by using the Basic Local Alignment Search Tool (BLAST, https://blast.ncbi.nlm.nih.gov/Blast.cgi), we verified that there were no identical sequences to four of them among all equine mitochondrial published data. The only exception was HT8, classified in I haplogroup, identical to the mtDNA control region from a Welsh pony (HQ439496).

Through the analysis of pairwise genetic distances between populations, we obtained a plot where IHD showed a very high variability within the breed, but a low Nei’s distance with the other populations here analysed, except for the CRC, which together with PER, showed the highest genetic distance from the other breeds (Fig. S2). Furthermore, BAR and HUD resulted being the most closely related to IHD, while CRC and BED were the most distant. By comparing all the breeds, we observed the lowest average number of pairwise differences within the Vyatskaya which confirmed the reduction in population size that involved this breed (Fig. S2). VYA is a West-Russian breed influenced by native Estonian horses brought by Novgorod colonists in the 14th century. In 1917 it was virtually extinct; some efforts to re-establish this breed were made after the Russian Revolution and it entered the Endangered List of the FAO in 2007 (http://www.fao.org/dad-is).

**Figure 3:**
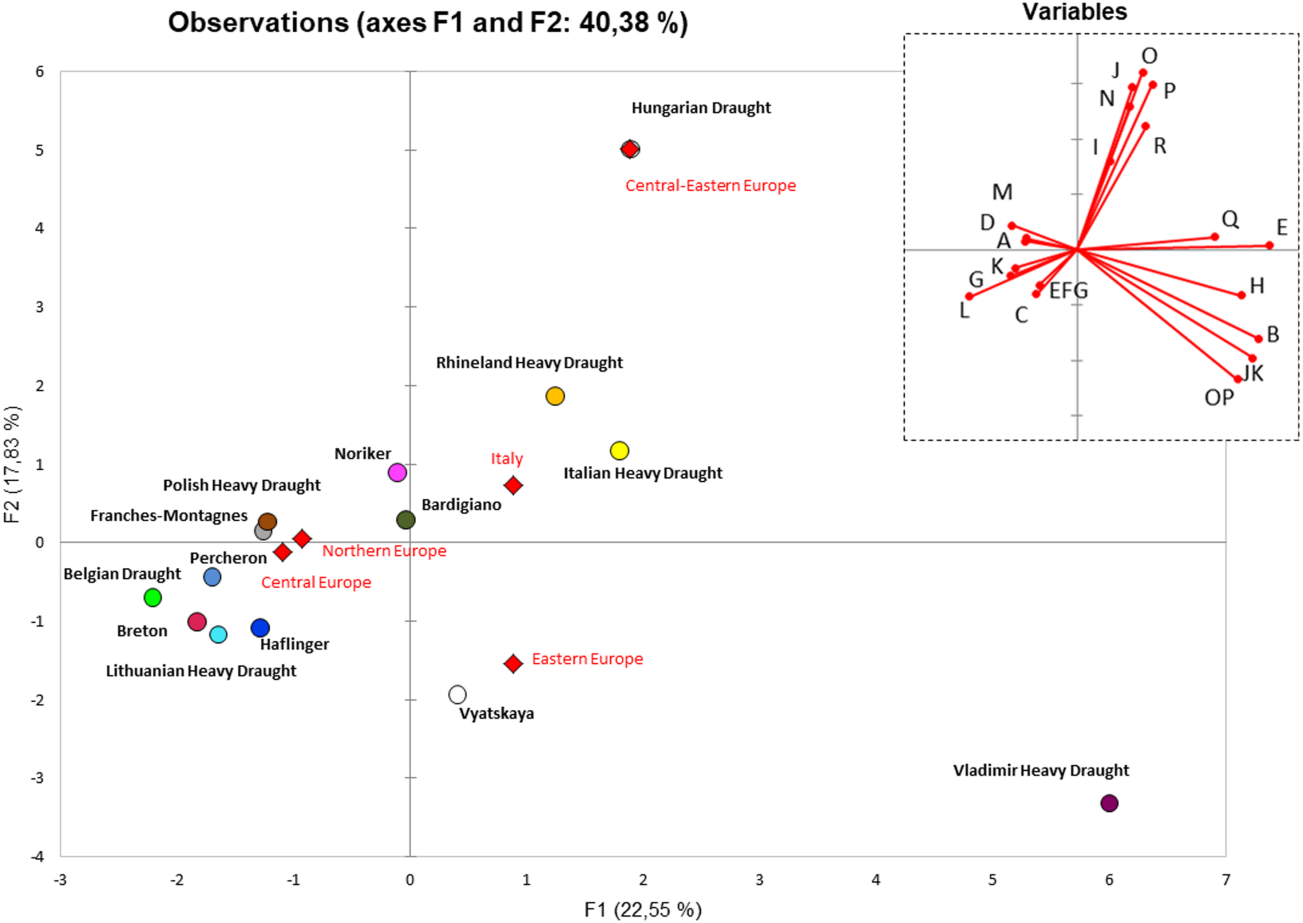
PCA plot representing the genetic landscape of European Heavy Horse breeds here considered, based on haplogroup frequencies from control-region data. All Italian Heavy Draught samples (*n* = 52 from this study; *n* = 27 from Bigi, Perrotta & Zambonelli (2014)) were grouped. Unclassified samples were excluded from the analysis. The right upper plot represents the contribution of each haplogroup to the first and second PC (projections of the axes of the original variables). Diamonds represent the centroids of breeds from the same macrogeographic area.

In order to summarize the information embedded in the haplogroup frequencies, we performed a principal component analysis (PCA). After reducing the variables (haplogroups) to PCs, we reported the coordinates of the observations for the European Heavy Draught Horse breeds in a two-dimensional graph based on haplogroup frequencies from control-region data (Fig. 3). We observed that although the Hungarian Draught was in the same quarter of RHD and IHD, not far from BAR, it stayed in an outlier position due to the highest frequency of J, O, and P haplogroups (Table S4, Fig. 3). This distribution was substantially unvaried when we included a wider range of horse breeds for a total of 2229 mtDNA control region sequences belonging to 54 European breeds (Fig. S3 and Table S5).

In order to better explore the peculiarities of IHD samples and to verify the clustering degree, we repeated the PCA analysis by considering single samples so that each individual sequence was represented by a point (Fig. S4). The overall plot confirmed the closeness of IHD to both HUD and BAR as shown by the centroids of each breed. At the same time, the figure shows the high genetic variability within the considered horse breeds and highlights the lack of clustering within the breed. The mtDNA haplotypes were not preferentially distributed among breeds, thus confirming the impossibility to employ the mitochondrial genome as an equine breed-specific marker.

Our findings pointed out that the ancestral local mares harbored a very high genetic diversity, verified the autochthonous origin of the breed (at least from the female perspective) and confirmed that the documented contribution of other European coldblooded breeds was restricted to stallions.

## Conclusions

Appropriate evaluation and characterization of animal populations both at phenotypic and genotypic levels are crucial steps in the development of conservation strategies. Most of the European Heavy Draught horses seem to derive from crossbreeding local mares and heavy draught stallions imported from other countries, and the genetic diversity of native horse breeds could be preserved only by considering also molecular data. For the first time, this study provides a phylogenetic assessment of the mitochondrial distribution within the Italian Heavy Draught Horse breed. The high mtDNA haplotype variability found in our IHD samples reflected the multiple maternal origins of the horses which underwent horse domestication (Achilli et al., 2012). Our results showed that samples were widely distributed in the network and shared many haplotypes with other European Heavy Draught Horse breeds. Although they were collected in a delimited area, four haplotypes were detected only in our samples and were not shared with other breeds, thus demonstrating a mitochondrial peculiarity that needs to be preserved.

These findings reflect the overall mtDNA legacy of the ancestral mares that were probably used at the initial stages of breeding selections a long time ago. Those mitochondrial lineages were preserved during the sex-biased breeding practices that often involved the intensive use of few selected external stallions (Wallner et al., 2013).

In conclusion, our study represents the first mitochondrial characterization of Italian Heavy Draught Horse breed sampled in Central Italy and can be considered a preliminary analysis for further investigations aiming to preserve the genetic peculiarities of local stocks.

##  Supplemental Information

10.7717/peerj.8996/supp-1 Figure S1 Median-Joining Network based on the control-region sequences of Italian Heavy Horses from this study (yellow) and Bigi, Perrotta & Zambonelli (2014) (black) The asterisk indicates the haplotype identical to ERS (Equine reference sequence; NC_001640.1).

10.7717/peerj.8996/supp-2 Figure S2 Plot of pairwise population genetic distances obtained by the concomitant analysis of European Heavy horses All Italian Heavy Draught samples ( *n* = 52 from this study; *n* = 27 from (Bigi, Perrotta & Zambonelli, 2014)) were grouped. Breed code as in Table 1. Breeds represented by three samples were excluded from the analysis.

10.7717/peerj.8996/supp-3 Figure S3 Two-dimensional region-based PCA plot obtained by including the available mtDNA control region data belonging to 54 European horse breeds The comparison between the Italian Heavy Draught (IHD) to a wider range of horses then considered in Fig. 3, based on haplogroup frequencies. Some breeds, such as Thoroughbred, were included as outgroup. Unclassified samples and breeds with less than four samples were excluded from the analysis. The red labels represent the centroids of breeds from each macrogeographic area.

10.7717/peerj.8996/supp-4 Figure S4 PCA plot representing the genetic landscape of European Heavy Horse breeds here considered, based on their haplotypes and mutation frequencies All Italian Heavy Draught samples ( *n* = 52 from this study; *n* = 27 from Bigi, Perrotta & Zambonelli (2014)) were grouped. Each dot represents a single sample. Diamonds represent the centroids of breeds. Colors reflect breeds as in legend.

10.7717/peerj.8996/supp-5 Table S1 The complete list of 52 Italian Heavy Draught horse samples collected from different farms of Central Italy

10.7717/peerj.8996/supp-6 Table S2 Control-region haplotypes and haplogroup classification of the European Heavy Draught horse breeds included in the analyses

10.7717/peerj.8996/supp-7 Table S3 Frequencies (%) of unique and shared haplotypes of mtDNA control region sequences (nps 15532-15738) between all European Heavy Draught Horse breeds here analysed

10.7717/peerj.8996/supp-8 Table S4 Haplogroup frequencies (%) distribution for each breed based on their mtDNA control regions

10.7717/peerj.8996/supp-9 Table S5 Source data for the PCA of European horse breeds Source data for the PCA of European horse breeds including haplogroup frequencies and breed’s geographic origin

10.7717/peerj.8996/supp-10 File S1 Raw data

10.7717/peerj.8996/supp-11 Supplemental Information 1 English editing ceritification

## Additional Information and Declarations

### DNA Deposition

The following information was supplied regarding the deposition of DNA sequences: The sequences are available at GenBank: MN719408 to MN719459.

### Data Availability

The following information was supplied regarding data availability: Sequences are available in File S1.
