# Supplementary figures and images for: Mitochondrial DNA variation in the Italian Heavy Draught Horse
Source: PeerJ. 2020 May 15;8:e8996. doi: 10.7717/peerj.8996 (PMC7233276; doi:10.7717/peerj.8996)

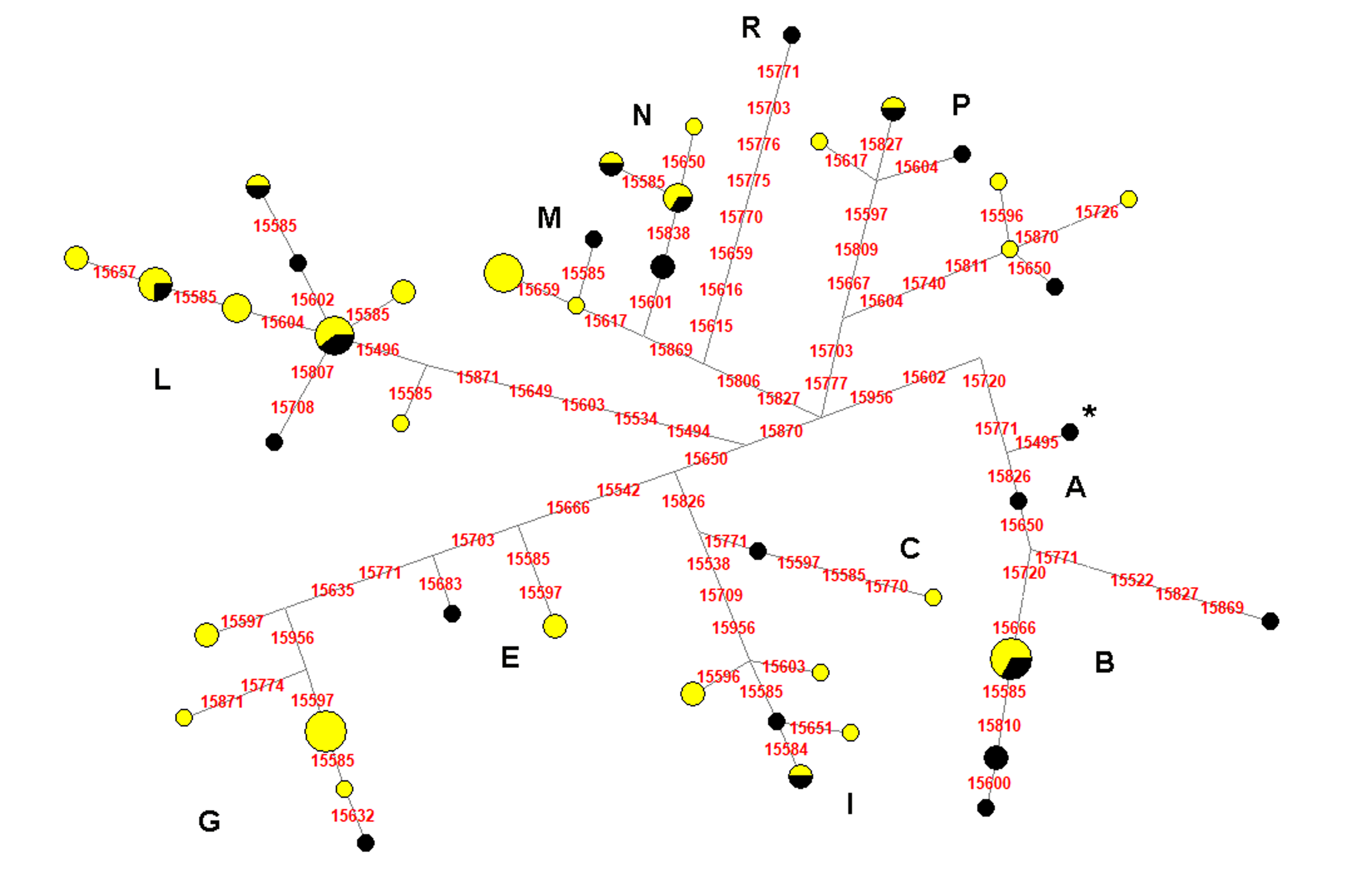

Supplement: Figure S1 — The asterisk indicates the haplotype identical to ERS (Equine reference sequence; NC_001640.1). [file peerj-08-8996-s001.png]

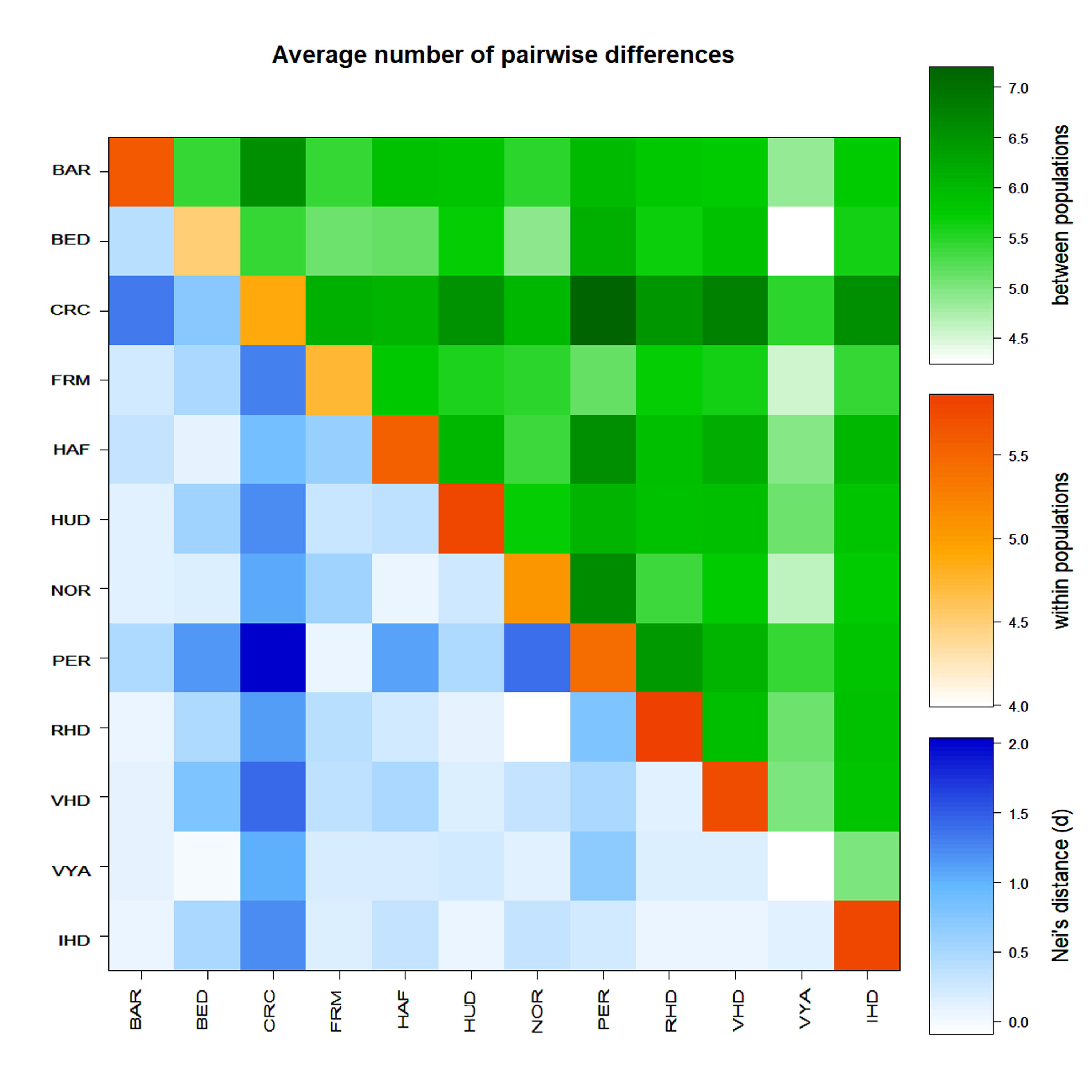

Supplement: Figure S2 — All Italian Heavy Draught samples ( n = 52 from this study; n = 27 from (Bigi, Perrotta & Zambonelli, 2014)) were grouped. Breed code as in Table 1. Breeds represented by three samples were excluded from the analysis. [file peerj-08-8996-s002.png]

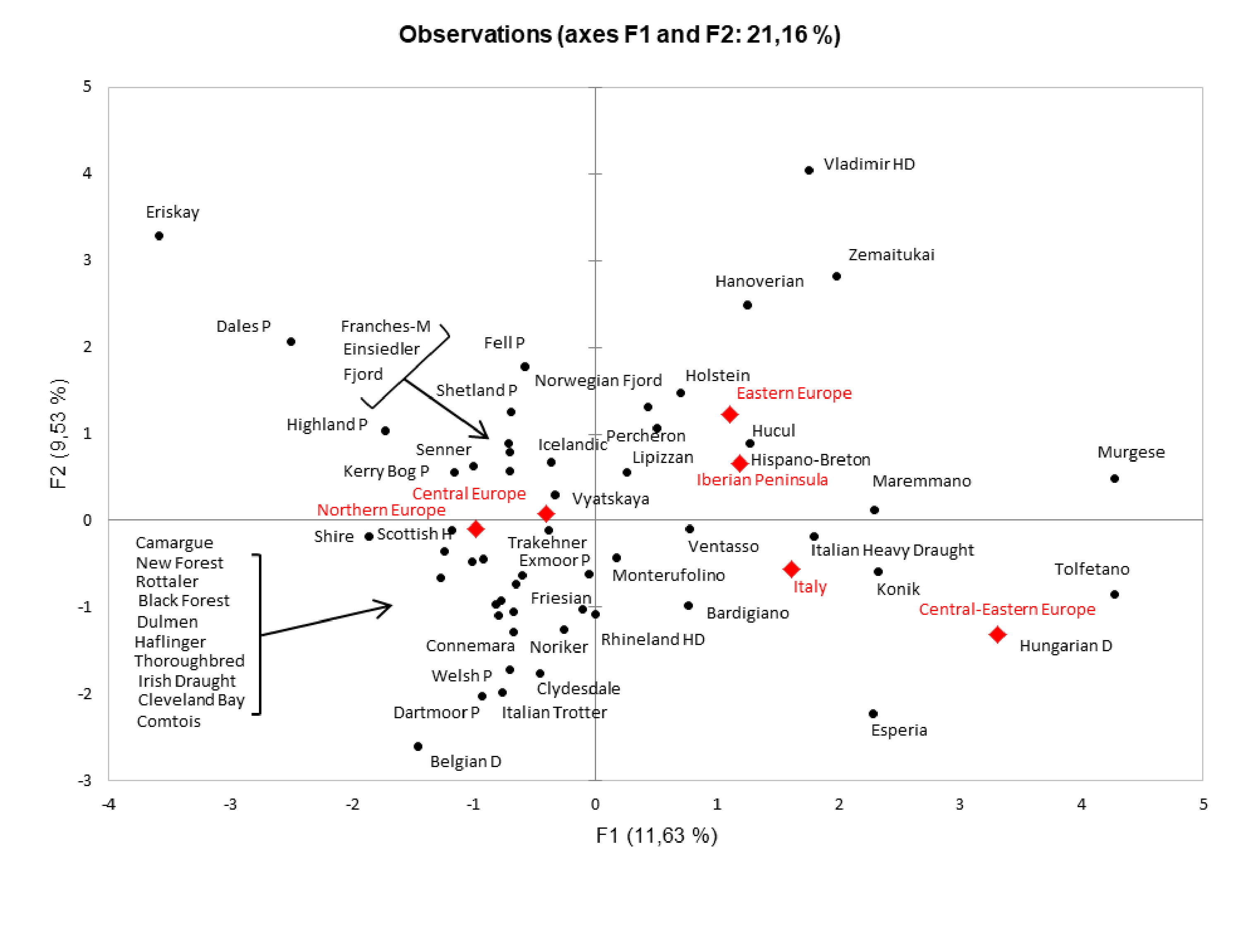

Supplement: Figure S3 — The comparison between the Italian Heavy Draught (IHD) to a wider range of horses then considered in Fig. 3, based on haplogroup frequencies. Some breeds, such as Thoroughbred, were included as outgroup. Unclassified samples and breeds with less than four samples were excluded from the analysis. The red labels represent the centroids of breeds from each macrogeographic area. [file peerj-08-8996-s003.png]

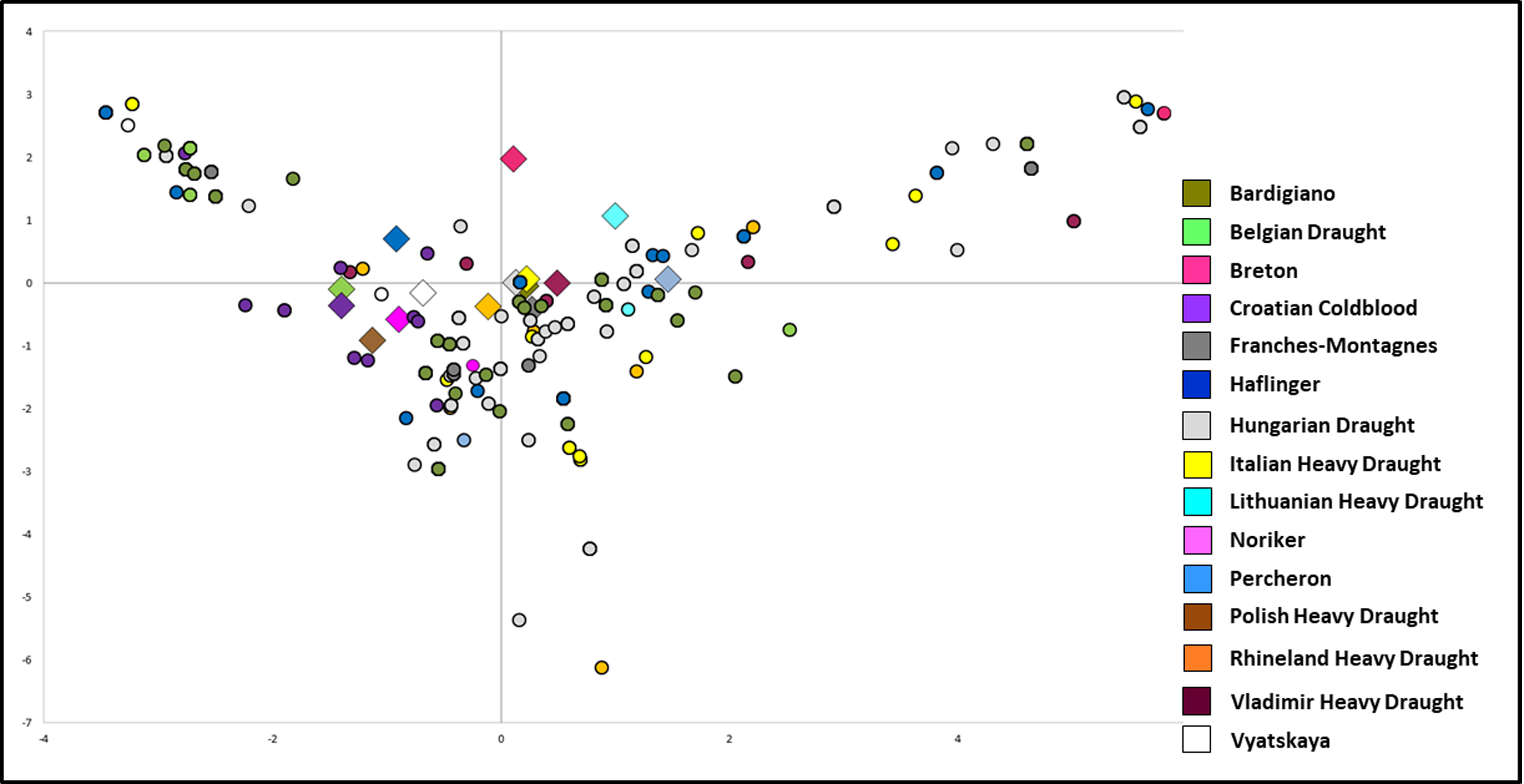

Supplement: Figure S4 — All Italian Heavy Draught samples ( n = 52 from this study; n = 27 from Bigi, Perrotta & Zambonelli (2014)) were grouped. Each dot represents a single sample. Diamonds represent the centroids of breeds. Colors reflect breeds as in legend. [file peerj-08-8996-s004.png]
