# Supplementary material for: Mitochondrial DNA variation in the Italian Heavy Draught Horse
Source: PeerJ. 2020 May 15;8:e8996. doi: 10.7717/peerj.8996 (PMC7233276; doi:10.7717/peerj.8996)
Supplement: Supplemental Information 1 [file peerj-08-8996-s011.pdf]

<u>EDITING CERTIFICATION</u>

This is to certify that the manuscript with the title " **Mitochondrial DNA variation in the Italian Heavy Draught Horse** " to be submitted by **Hovirag Lancioni**, **Irene Cardinali**, **Andrea Giontella**, **Maria Teresa Antognoni** and **Arianna Miglio** has been edited for proper English language, grammar, punctuation, spelling, and overall style by Professor Paul Quattrone (EFL Academic Teacher Coordinator) and Alda Quattrone (Bachelor of Science in Animal Husbandry).
Therefore, we guarantee the quality of the English language in this manuscript.
Neither the research content nor the author's intentions were altered in any way during the editing process.

Perugia, 16th March 2020

Paul Quattrone

Alda Quattrone
